# Plasticiser-Free 3D Printed Hydrophilic Matrices: Quantitative 3D Surface Texture, Mechanical, Swelling, Erosion, Drug Release and Pharmacokinetic Studies

**DOI:** 10.3390/polym11071095

**Published:** 2019-06-28

**Authors:** Zara Khizer, Muhammad R. Akram, Rai M. Sarfraz, Jorabar Singh Nirwan, Samia Farhaj, Maria Yousaf, Tariq Hussain, Shan Lou, Peter Timmins, Barbara R. Conway, Muhammad Usman Ghori

**Affiliations:** 1 Department of Pharmacy, School of Applied Sciences, University of Huddersfield, Huddersfield HD1 3DH, UK; 2 College of Pharmacy, University of Sargodha, Sargodha 40100, Pakistan; 3 System Engineering Department, Military Technological College, Muscat 111, Oman; 4 The Wolfson Centre for Bulk Solid Handling Technology, University of Greenwich, London SE10 9LS, UK; 5 School of Computing and Engineering, University of Huddersfield, Huddersfield HD1 3DH, UK

**Keywords:** 3D printing, hot melt extrusion, hydroxypropyl methyl cellulose (HPMC), swelling, erosion, drug release, pharmacokinetics, Young’s modulus, 3D surface texture

## Abstract

Hydroxypropyl methyl cellulose, HPMC, a hydrophilic polymer, is widely used for the development of extended release hydrophilic matrices and it is also considered as a good contender for the fabrication of 3D printing of matrix tablets. It is often combined with plasticisers to enable extrusion. The aim of the current project was to develop plasticizer-free 3D printed hydrophilic matrices using drug loaded filaments prepared via HME to achieve an in vitro (swelling, erosion and drug release) and in vivo (drug absorption) performance which is analogous to hydrophilic matrix tablets developed through conventional approaches. Additionally, the morphology of the printed tablets was studied using quantitative 3D surface texture studies and the porosity calculated. Filaments were produced successfully and used to produce matrix tablets with acceptable drug loading (95–105%), mechanical and surface texture properties regardless of the employed HPMC grade. The viscosity of HPMC had a discernible impact on the swelling, erosion, HPMC dissolution, drug release and pharmacokinetic findings. The highest viscosity grade (K100M) results in higher degree of swelling, decreased HPMC dissolution, low matrix erosion, decreased drug release and extended drug absorption profile. Overall, this study demonstrated that the drug loaded (glipizide) filaments and matrix tablets of medium to high viscosity grades of HPMC, without the aid of plasticisers, can be successfully prepared. Furthermore, the in vitro and in vivo studies have revealed the successful fabrication of extended release matrices.

## 1. Introduction 

Presently, pharmaceutical industries utilise well established methods for the fabrication of tablets with predetermined dose, size and shape [1]. Although the current methods are common and cost-effective, they offer little opportunity for personalisation and on-demand manufacturing as the change in size, shape and dose of a tablet require alterations at each manufacturing step and retooling of tabletting machines [2,3,4]. Consequently, a technology capable of producing dosage forms, with a variety of strengths, such as three dimensional (3D) printing, is required to accomplish the personalised therapeutic needs of individuals [5]. This technology has enabled cost-effective fabrication of individualised dosage forms and has potential to develop personalised pharmaceutical products [3,5]. The recent availability of Spritam®, although it is not personalised, in the United States has proven the industrial and commercial feasibility of this technology, yet there are some regulatory hurdles [2,6,7].

Various 3D printing technologies are used in practice including stereolithography, selective laser sintering, powder-based printing and fused deposition modelling (FDM). FDM is one of the most common with significant potential for achieving personalised dosage forms as it can be used to deposit a large range of doses and multiple drugs into solid dosage forms of almost any shape [8]. FDM printing involves feeding of filaments into the printer, melting and layer-by-layer deposition that then solidifies resulting in the desired dosage form [9,10]. The amount of drug incorporated into the dosage form can thus be tailored according to the weight and physiological needs of the patient [11]. Research has focused on using already established materials for filament production, although there are no commercial products available yet [6,11,12,13,14,15,16,17,18,19].

Additional work is required to understand the potential of the process and how any variation or modification will affect performance and functionality of the products. Many research groups have attempted to develop filaments using individual or a combination of pharmaceutical polymers along with other excipients (plasticisers) by hot melt extrusion (HME) [15,16,18,19]. HME coupled with FDM has several advantages over conventional tablet manufacturing including an increased capacity for drug loading. A study carried out by Pietrzak et al. (2015) [20] combined FDM 3D printing with HME in an attempt to extend the range of polymers that can be used and achieve higher drug loading. They demonstrated the feasibility of printing immediate and extended theophylline caplets based on cellulosic or methacrylic polymeric filaments with 50% w/w drug loading. Moreover, numerous other studies have demonstrated the adaptability of various polymers with FDM including: Acrylonitrile butadiene styrene [17], poly (ε-caprolactone [18], ethyl cellulose [19], ethyl vinyl acetate [21], hydroxypropyl cellulose [20,22], hydroxypropyl methylcellulose acetate succinate [23] and hydroxypropyl methyl cellulose, HPMC [2,24,25]. 

Among these polymers, HPMC is the most widely used for developing drug-loaded filaments and has previously been used to fabricate 3D printed tablets [2,24]. Polymers are often combined with plasticisers to facilitate their extrusion. The inclusion of plasticiser may impact the glass transition (*T_g_*) temperature, as described by Bruce et al. [26] Polyethylene glycol, triacetin and triethyl citrate are commonly used plasticisers [15,16,19] however, various studies have demonstrated that the presence of these aforementioned plasticisers either alone or in pharmaceutical formulations may have an impact on gastrointestinal motility, affecting gastric and intestinal transit times [27,28,29]. Both gastric and intestinal transit play an important role during drug absorption. Thus, their presence especially in hydrophilic matrices could have an impact on their functional performance [30]. Furthermore, these above findings, related to plasticiser induced gastric motility become more significant for individuals suffering from chronic diseases, for example diabetes and cardiac disorders, where patients have to be on life-long pharmacotherapy and excessive exposure to plasticisers might lead to drug absorption complications.

In order to be used in the FDM process, the drug-loaded filaments need to be mechanically robust as soft or brittle filaments will be damaged by the feeding gear. Mechanical studies such as a three-point bending test can be used to characterise material properties [24]. Additionally, using FDM for tablet production can lead to products with seam-line issues and unsatisfactory seam-line issues, affecting both appearance and possibly performance [31]. Porosity is an important characteristic of hydrophilic matrices as higher overall porosity leads to rapid penetration of liquid molecules in comparison to lower overall porosity thus impacting the swelling behaviour [32]. In order to address these challenges, we proposed to develop plasticizer-free 3D printed hydrophilic matrices using drug loaded filaments prepared via HME to achieve an in vitro (swelling, erosion and dug release) and in vivo (drug absorption) performance which is analogous to hydrophilic matrix tablets developed through conventional approaches. Additionally, to evaluate the tablet appearance issues, porosity and quantitative 3D surface texture studies were carried out using mercury intrusion porosimetry and white light optical profilometry, respectively. Glipizide which is an anti-diabetic drug of the sulfonylurea class indicated to treat type-2 diabetes was used as a model drug. Glipizide is a weak acid (pKa = 5.9), practically insoluble in water and acid, and is highly permeable (biopharmaceutics classification system, BCS II). It appears to be an effective insulin secretagogue which reaches a peak plasma concentration within 1–3 h after a single oral dose with an elimination half-life of about 2–4 h [33]. Such rapidly absorbed drugs having fast elimination rates with short half-life make it suitable candidate to be considered for sustained delivery.

## 2. Materials and Methods 

### 2.1. Materials

Glipizide was used as the model drug and purchased from TCI Europe (Zwijndrecht, Belgium). HPMC polymer of different grades with increasing molecular size/weight (Methocel® K4M, K15M and K100M) was kindly provided by Colorcon Ltd (Dartford, UK). Specifications of different HPMC grades are listed in Table 1.

### 2.2. Preparation of Filaments 

The glipizide loaded filaments of each HPMC grade (K4M, K15M and K100M) were developed from the premixed HPMC: glipizide (2.5% w/w) powder mixtures using a single screw extruder (Noztek® Pro pellet and powder extruder, Sussex, UK). The extrusion temperature and nozzle diameter were 155 °C and 1.75 mm, respectively. Once the filaments were extruded, they were stored in a desiccator at room temperature until further use.

### 2.3. Physicochemical Characterisation of Filaments 

#### 2.3.1. Determination of Drug Loading

A sample (0.2 g) of the glipizide-loaded filament of each polymer grade was placed in a 1 liter methanol:water (1:1) solvent mixture under magnetic stirring until complete dissolution. Liquid samples were then filtered and the dissolved glipizide content was determined using Jenway 6405 UV spectrophotometer, Staffordshire, UK, all the measurements were carried out in triplicate. 

#### 2.3.2. Differential Scanning Calorimetry (DSC)

Differential scanning calorimetery (Mettler Toledo SC 821, Mettler-Toledo Ltd., Leicester, UK) was used to study all the powder samples (plain drug, polymers, and their extruded filaments). Briefly, 5–10 mg of the samples were placed in standard aluminium pans and experiment was run under nitrogen environment (50 mL/min). To assess the physical form of the glipizide within the polymeric matrix a heat scan from 25–250 °C was applied to the samples at 10 °C/min.

#### 2.3.3. Thermogravimetric Analysis (TGA)

TGA was performed using Mettler Thermobalance TG50 (Mettler-Toledo Ltd., Leicester, UK). Open alumina crucibles were used to analyse all the plain powder samples and filaments (5–10 mg). Samples were heated from 25–250 °C at 10 °C/min heating rate and nitrogen was used as purge gas with a flow rate of 50 ml/min.

#### 2.3.4. X-ray Powder Diffraction (XRPD)

Samples (plain drug and polymers) and filaments were characterised using a D2-Phase X-ray diffractometer (Bruker UK Ltd., Coventry, UK) equipped with a CuKɑ radiation source at 30 KV voltage and 10 mA current. Diffraction patterns were obtained in the 2 theta (θ) range of 5°–100° using 0.02 step sizes. 

#### 2.3.5. Scanning Electron Microscopy (SEM)

The morphology of all the drug loaded filaments was observed using scanning electron microscopy (SEM) [34]. Briefly, samples were mounted onto stubs using double-sided adhesive tape and were sputter-coated with gold/palladium (80:20) for 60 seconds using a Quorum SC7620 Sputter Coater (Quorum Technologies, Laughton, UK) and were examined photometrically using Jeol JSM-6060CV, Jeol Inc., Peabody, MA, USA.

#### 2.3.6. Mechanical Testing 

All the extruded HPMC: Glipizide filaments were cut in to 10 cm pieces and were placed on a TA-95N 3-point bend probe set which was attached to a TA-XT2i texture analyser (Stable Micro Systems Ltd, Surrey, UK) for mechanical characterisation. The moving speed of the blade was 5 mm/s until it reached 15 mm under the sample. The mechanical data was collected and analysed using Exponent® software (Stable Micro Systems Ltd, Surrey, UK).

### 2.4. Fabrication of 3D Printed Glipizide Matrix Tablets 

A cylindrical tablet was designed (5 mm in diameter with a 2.5 mm height) using SolidWorks® version 2015, then converted into *stl.* (stereolithographic) format. The drug loaded filaments were loaded into MakerBot replicator mini (MakerBot Inc., New York, NY, USA) using a constant infill density (100%) and pattern (linear). The 3D printing parameters used in the preparation of matrices are listed in Table 2.

### 2.5. Characterisation of 3D Printed Matrices

#### 2.5.1. Geometrical, Porosity and Morphological Assessment of Matrices

A digital calliper was used to determine the diameter and thickness of the tablets and the porosity of the 3D printed matrices was determined using mercury intrusion porosimetry (Auto Pore IV 9500, Micrometrics, Norcross, GA, USA) [35]. The surface morphology of the printed matrices was assessed using Jeol JSM-6060CV, Jeol Inc., Peabody, MA, USA.

#### 2.5.2. Determination of Tablet Strength 

A general material testing machine (Testometric M500–50 CT, Testometric Company Ltd., Rochdale, United Kingdom) was used for hardness testing of the matrix tablets. The printed tablets were placed diametrically and force was applied by the movement of upper punch at the rate of 10 mm/min until the tablet breaks. Ten tablets from each group of matrix tablets were tested. 

#### 2.5.3. Determination of Tablet Friability

10 tablets from each group were weighed and placed in the tablet friability testing instrument (PTF 20E, Pharmatest, Hainburg, Germany). The drum of the friability instrument was then rotated at 20 rpm for 5 min and tablets were re-weighed. The friability was calculated in terms of weight loss and expressed as a percentage of the original weight of the tablets. 

#### 2.5.4. 3D Nanoscale Surface Texture Analysis

Surface texture of 3D printed matrix tablets was examined using Talysurf CCI 3000 optical 3D surface profiler and the method was adopted as previously described by Diryak et al., 2018 and Khizer et al., 2019. ^25,36^ Briefly, the tablet was fixed on a stainless steel wafer (3 × 3 cm) using double-sided transparent tape and a 800 × 800 μm region of each tablet was scanned and 3D quantitative surface texture parameters were calculated using MATLAB 2017 (The Math Works, Inc. Natick, MA USA) [25].

#### 2.5.5. Swelling Studies

Swelling studies were carried out using USP apparatus I, SR II 6-flask (Hanson Research, Chatsworth, CA, USA) at 75 rpm at 37 °C. The pre-weighed 3D printed matrix tablets (*W_i_*) were immersed in 900 mL swelling media for 2 h in simulated gastric fluid, SGF pH1.2 and 22 h in simulated intestinal fluid, SIF pH 6.8. The previously weighed baskets, containing hydrated matrix tablets, were removed at different time points, lightly blotted with 125 mm filter paper (Whatman®, Maidstone, Kent, UK) to remove excess liquid, reweighed (*W_s_*) and were rapidly replaced back into the swelling media in dissolution apparatus. The mean weight was determined for each formulation and degree of swelling (S) was calculated by using Equation 1) [36,37,38,39].

(1) $$S=\frac{W_{s}-W_{i}}{W_{i}}\times 100$$

where *W_i_* and *W_s_* are the initial dry and swollen weight of the matrix tablet, respectively, at immersion time (t) in the swelling media. The degree of swelling was determined from the mean of three replicates and presented as degree of swelling (S, %) against time (t).

#### 2.5.6. In Vitro Glipizide Dissolution Studies

The in vitro glipizide dissolution studies were carried out using USP I basket apparatus where the basket rotation speed and temperature were maintained at 75 rpm and 37 °C, respectively. The pre-weighed 3D printed matrix tablets were immersed in 900 mL dissolution media for 2 h in simulated gastric fluid (pH 1.2) and then 22 h in simulated intestinal fluid (pH 6.8). At predetermined times, 5 mL aliquots were drawn from the dissolution apparatus and replaced with 5 mL of fresh dissolution media. The released glipizide was quantified using UV-spectroscopy at ʎ _max_ (225 nm) and glipizide mass was determined using a standard calibration curve, all the measurements were carried out in triplicate.

#### 2.5.7. HPMC Dissolution and Overall Erosion Studies 

To quantify dissolved HPMC, a previously described procedure was adapted to enable analysis of multiple samples) [40,41,42,43]. In the modified method, 20 µL of 5% v/v phenol was added to 20 µL of liquid sample containing HPMC previously placed into a microplate followed by mixing (5 min) using shaking plate. Once these were mixed, 100 µL of H_2_SO_4_ was added to each well and subjected to mixing again for 5 min. The solutions were then incubated for 15 min at room temperature (20–25 °C) before the UV absorbance was read at 488 nm using a microplate reader and dissolved HPMC was quantified using a standard calibration curve for each grade of HPMC. Moreover, overall matrix erosion was calculated by simply adding the quantities of drug and HPMC dissolved at a specified time point.

### 2.6. Pharmacokinetic Studies 

#### 2.6.1. Animal Housing and Handling

White albino rabbits weighing 2.20–2.50 kg were used in these experiments which were further divided into four groups, group I–IV (five rabbits per group). All rabbits were housed individually in cages under environmentally controlled conditions (25 ± 2 °C; 50 ± 5% relative humidity). All the rabbits had free access to food and water except for during the final 24 h before the experiments. The study protocol was approved by the Pharmacy Research Ethics Committee (PREC) at the University of Sargodha, Pakistan (UOS/PERC/101).

#### 2.6.2. In vivo Experiments 

A single-dose pharmacokinetic study was carried out and rabbits of group I were administered glipizide oral solution (2.5 mg in 20 mL of deionised water) and 3D printed glipizide matrix tablets of K4M, K15M and K100M (100 ± 1.5 mg containing 2.5% w/w glipizide) were administered to the rabbits of group II, III and IV, respectively. At different time intervals (0, 15, 30, 60, 90, 120, 150, 180, 240, 300, 360, 420, 480, 540, 600, 660, 720, 1080, 1440 minutes), 1 mL of blood samples were collected from the marginal ear vein into heparinised tubes. The collected blood samples were then centrifuged for 15 min at an ambient temperature. After centrifugation the plasma layer was separated and was stored at –20 °C until analysed. 

#### 2.6.3. Quantification of Glipizide in Plasma

0.5 mL of the rabbit plasma was acidified with 150 µL 0.5 M HCl and vortex-mixed for 2 min. This acidified plasma was then further mixed with benzene for 3 min and centrifuged for 15 min at 4400 rpm. The organic layer was separated and dried at 35 °C under a nitrogen stream [44]. Once dried the residues were dissolved in 200 µL methanol and filtered. The filtrate (20 μL) was then injected into the HPLC consisting of reverse phase C-18 column (Phenomenex Ltd, Torrance, CA, USA). The mobile phase used was acetonitrile: methanol: H_2_O (40:10:50 % v/v) at a flow rate of 1mL/min and glipizide was detected using UV at a wavelength of 275 nm. 

#### 2.6.4. Determination of Pharmacokinetic Parameters

PKSolver program, an add-in macro for Microsoft Excel®, was employed for the calculation of the different pharmacokinetic parameters) [45].

## 3. Results and Discussion 

### 3.1. Development and Characterisation of Filaments

**Figure 1:**
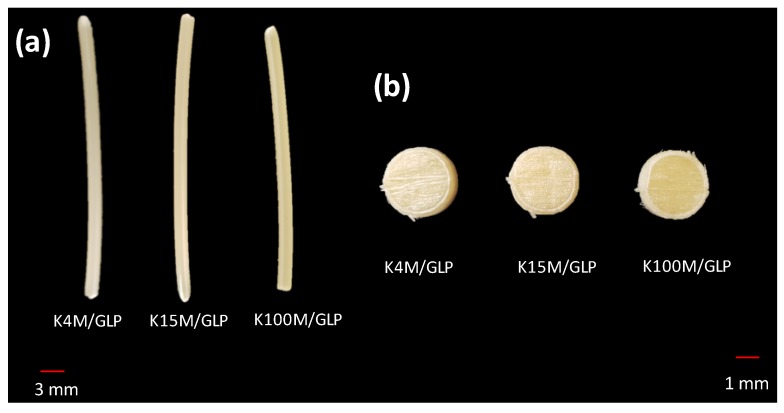
(**a**) Hot melt extruded filaments and (**b**) 3D printed hydrophilic matrices.

**Figure 2:**
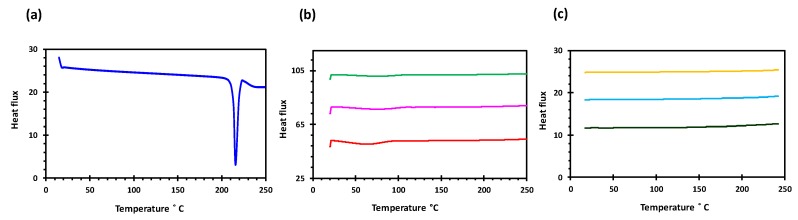
DSC profiles of (**a**) glipizide, (**b**) HPMC (K4M, K15M and K100M) and (**c**) K4M/GLP, K15M/GLP and K100M/GLP filaments.

**Figure 3:**
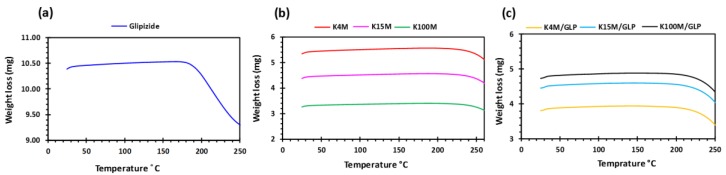
TGA profiles of (**a**) glipizide, (**b**) HPMC (K4M, K15M and K100M) and (**c**) K4M/GLP, K15M/GLP and K100M/GLP filaments.

**Figure 4:**
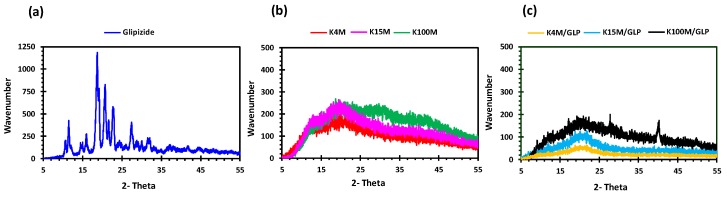
XRD profiles of (**a**) glipizide, (**b**) HPMC (K4M, K15M and K100M) and (**c**) K4M/GLP, K15M/GLP and K100M/GLP filaments.

**Figure 5:**
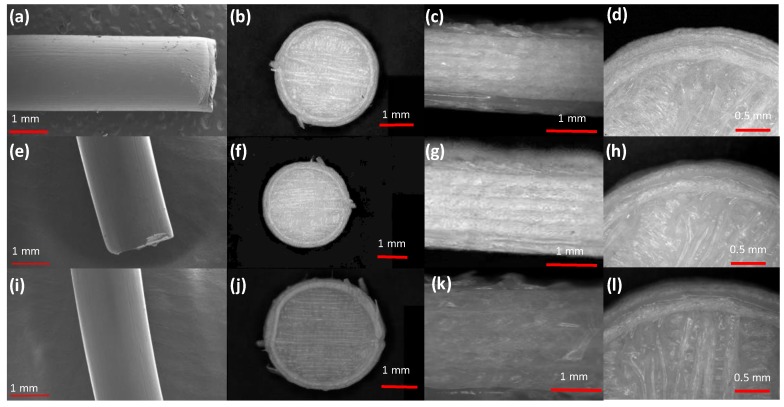
SEM micrographs of (**a–d**) K4M based filament and matrix tablet (a = filament, b = whole matrix tablet, c = side view of matrix tablet and d = surface view showing edges), (**e**-**h**) K15M based filament and matrix tablet (e = filament, f = whole matrix tablet, g = side view of matrix tablet and h = surface view showing edges) and (**i–l**) K100M based filament and matrix tablet (i = filament, j = whole matrix tablet, k = side view of matrix tablet and l = surface view showing edges).

Glipizide loaded HPMC filaments were successfully extruded without the aid of plasticiser using HME (Figure 1a). The glipizide loading (2.5% *w/w*) in extruded filaments of each grade (K4M, K15M, and K100M) was within the pharmacopoeial assay limit. Notably, in the present research higher glipizide loading was achieved in comparison to previously reported results) [46] (Table 3). Thermal analysis (DSC and TGA) and XRD were conducted on powdered drug sample and extruded filaments. Glipizide powder shows a sharp endothermic melting peak at 214.5 ˚C, demonstrating its crystalline nature (Figure 2a). The lack of a melting peak in the DSC thermograms of HPMC displayed in Figure 2b indicated that the polymer is amorphous. DSC thermograms were also acquired for HPMC: glipizide filaments for all grades of HPMC to investigate any drug-polymer interactions and they also confirmed there was no crystalline drug evident in the extruded filament (Figure 2c). The TGA results showed that onset of degradation for both drug and filaments was above the operating temperature (170 ˚C) involved in the printing process (Figure 3). Furthermore, the crystalline structure of glipizide was confirmed by XRD of the drug which showed multiple high-intensity peaks (Figure 4a). XRD scans of different grades of HPMC filaments showed no peaks and scattering of x-rays was also not observed, highlighting the amorphous nature of the polymer (Figure 4b). Additionally, high intensity multiple peaks of glipizide were not seen in the XRD scan of drug loaded filaments which is likely due to the formation of a solid dispersion that might have masked the crystalline structure of glipizide (Figure 4c) [25]. Overall, XRD spectra of glipizide and drug loaded HPMC filaments were consistent with the DSC profiles, thus, both methods showed that glipizide has a crystalline structure, whereas HPMC and drug loaded filaments have amorphous nature. This may potentially enhance dissolution but could negatively impact stability. Additionally, SEM micrographs of HPMC filaments of different grades showed cylindrical shapes and smooth surfaces without any visible fine powder particles (Figure 5a,e,i).

For successful 3D printing, good mechanical properties are essential as brittle filaments have a tendency to crumble whereas too soft filaments may be squeezed aside by the feeding gear leading to printing failure. Hence, in this study a three-point bending test was used to evaluate the mechanical properties of the extruded filaments) [47,48] (Table 3). The force required to break the filaments was lowest for K4M filaments (1.9 ± 0.2 N) followed by K15M (2.2 ± 0.6 N) and K100M (2.3 ± 0.3 N). This trend was also seen in the stress required to break the filaments with K4M requiring 12.3 ± 1.1 MPa, K15M requiring 14.2 ± 1.2 MPa and K100M requiring 16.9 ± 1.6 MPa. The breaking distance for all the filaments extruded in this study was in the range of 4.9–5.2 mm. Moreover, Young’s modulus increased with polymer molar mass indicating increased polymer chain entanglement during the extrusion process. It has previously been reported that filaments with a breaking distance (toughness) of less than 1.5 mm were too brittle to be loaded into a 3D printer and were easily broken by the feeding gear [8]. All the filaments in this study demonstrated good mechanical properties (Table 3) and were successfully employed for the manufacturing hydrophilic matrices with constant infill design (linear) and density (100%) (Figure 1b). Interestingly, the mechanical performance of the extruded filaments are comparable to those of filaments developed by different researchers using plasticisers [24]. This interesting behaviour might be linked to plasticisation of the polymer by the comparatively low molar mass glipizide which has not only reduced its *Tg* but also helped its extrusion. It could plasticiser the polymer by reducing the secondary forces (hydrogen bonding and van der Waals forces) between the HPMC polymer chains by occupying intermolecular spaces. Therefore, the glipizide has changed the three-dimensional organisation of HPMC polymer chains which has reduced the overall energy required for molecular motion which has reduced the HME operating temperature well below the *Tg* of HPMC and has also improved the mechanical properties [49]. 

### 3.2. Development and Characterisation of 3D Printed Matrix Tablets 

All the glipizide loaded filaments produced 3D printed hydrophilic matrices, characterised as detailed in Table 4. There were no statistically significant differences in thickness, diameter and weight. In accordance with drug loading in the filaments, the drug loading in the 3D printed matrices was within the pharmacopoeial assay limit (95–105%) [50]. To evaluate the quality and investigate any structural defects of the printed matrices, porosimetry was carried out and matrices printed with K100M filaments had the lowest porosity (0.8 ± 0.1%) followed by K15M matrices (1.5 ± 0.2%) and K4M matrices (2.2 ± 0.2%), Table 4. The breaking strength of the tablets (N) was also determined. In comparison to traditional tableting press, the physical properties of tablets can be controlled by compression forces where 4 kg is the minimum breaking force value. Measured breaking force measurements for 3D printed hydrophilic matrices exceeded the accepted range [85.2–93.8 N (8.7–9.6 kg)] for solid tablets [9]. The 3D printing process does not involve compression forces so it is obvious that this parameter does not exist to manipulate the hardness of 3D printed tablets. It is evident that these printed tablets are quite robust and can bear a reasonable amount of rough handling which is evident from the friability results, Table 4. SEM micrographs of 3D printed HPMC matrices shows that they are not smooth due to the printing pattern of FDM (Figure 5b,f,j). The layering pattern on the surfaces of the tablets is visible in the top-view SEM images of the tablets. Also, the layer by layer approach of the FDM technique can be seen in the side-view SEM images of the tablets (Figure 5c,g,k). Although the surface of the tablets is not smooth compared to compressed tablets [39], however, the images confirm successful extrusion of filaments during tablet printing (Figure 5d,h,l).

**Figure 6:**
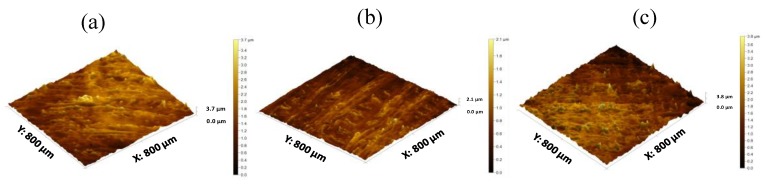
3D surface texture images (**a**) K4M (**b**) K15M and (**c**) K100M based matrix tablet.

Surface texture analysis of 3D printed tablets was conducted using a profilometer Figure 6a–c. Multiple surface texture parameters were calculated including amplitude, spacing, volume and hybrid parameters as presented in Table 5. Overall, these parameters displayed a spiked peak height distribution (Sku > 3) indicating the presence of inordinately high peaks and/or deep valleys and a predominance of valley structures (Ssk < 0) on the surface of the matrix tablets of all three grades of HPMC. Additionally, the surface roughness (Sa), density of peaks per unit area (Sds) and maximum height (sum of height of highest peak and height of deepest valley; Sz) were highest in K4M matrices followed by K100M and K15M, although the highest peak was present in the K100M matrix (Sp). The texture aspect ratio (Str) also followed the same trend with the highest in K4M followed by K100M and K15M matrices; however, all texture aspect ratios indicated strong isotropy (Str > 0.5). In addition, volume parameters, which represent the volume of space contained by the surface from a plane at a height corresponding to a chosen material ratio level to the lowest valley (vv and vvv), were highest in K4M matrix tablets followed by K100M and K15M, although the differences in vv were statistically insignificant. This trend was expected due to the deepest valley being found in K4M and the shallowest valley being found in K15M matrix tablets. On the other hand, material volume (vm), which represents the volume of space contained by the surface from a plane at a height corresponding to a chosen material ratio level to the highest peak, was greatest in K100M matrix tablets followed by K4M and K15M. This was anticipated due to the highest peak being found in K100M matrices. Overall, it can be concluded that the 3D printed matrix tablets were considerably rougher than the conventional compressed tablets, and that roughness is not directly related to polymer viscosity or chain length. 

### 3.3. Swelling, Erosion, HPMC Dissolution and Drug Release Studies

#### 3.3.1. Swelling and HPMC Dissolution Studies

**Figure 7:**
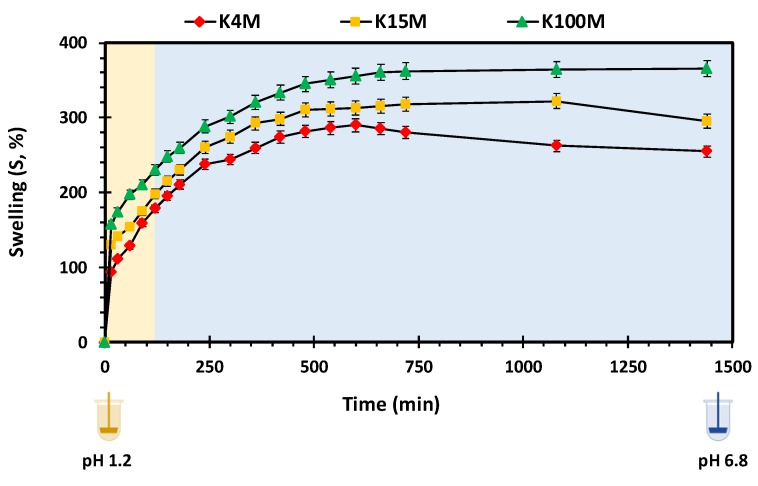
Swelling vs. time profile of 3D printed HPMC matrix tablets.

**Figure 8:**
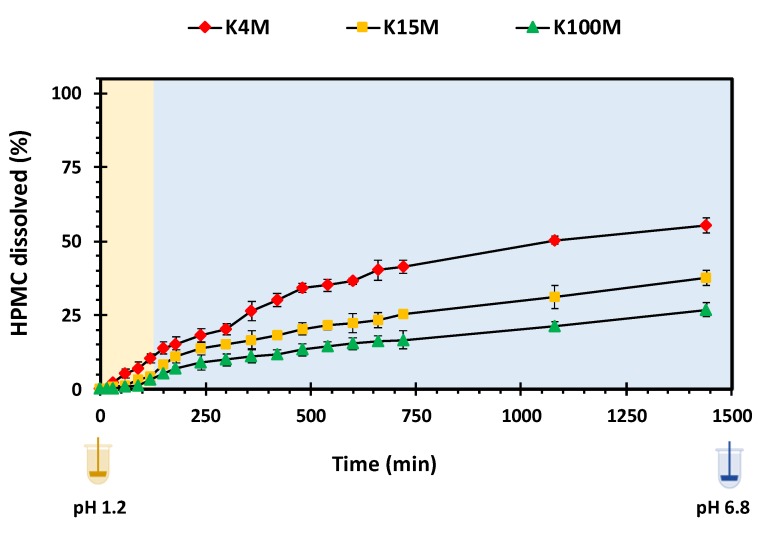
HPMC dissolution vs. time profile of 3D printed HPMC matrix tablets.

Polymer swelling is an important property in controlling drug release from hydrophilic matrices [51]. Figure 7 displays the swelling profiles of all types of 3D printed HPMC based glipizide matrices with respect to time. It is apparent that there is a direct relationship between the amount of swelling and viscosity (molecular size) of the polymer. In acidic pH, the maximum swelling achieved by K4M, K15M and K100M matrices was nearly 180%, 200% and 230%, respectively. Within 720 min (12 h), the maximum swelling achieved by K4M matrices was more than 250%. K15M matrices swelled by 300% whereas the highest amount of swelling was observed in K100M matrices with more than 350%. After achieving maximum swelling, a slight decline in swelling was observed with K4M and K15M matrices which is attributed to matrix erosion. The relationship between the molecular size (viscosity) of HPMC and extent of swelling may be explained due to the fact that higher molecular size polymers exhibit increased liquid uptake resulting in rapid swelling of the polymer. This was also supported by the rate of swelling, *K_w_*, obtained by fitting the data to a swelling kinetic model described by Vergnaud [52] as shown in Table 6. The R^2^ values displayed in the table (0.97-0.99) indicate that the data was well described by the Vergnaud mathematical model and, as an *n* value <0.5 is indicative of a diffusion-controlled mechanism, it can be deduced that swelling by the 3D printed matrix tablets follows a diffusion-controlled mechanism. Figure 8 shows the percentage of HPMC dissolved over time. It can be seen that the HPMC dissolution increased over time but declines with an increase in viscosity of the polymer. This is because high viscosity HPMC matrices form a more resilient and thicker gel on the matrix surface. The highest percentage of HPMC dissolved was observed with K4M matrices and the lowest was observed with K100M matrices. 

#### 3.3.2. Drug Release Studies

**Figure 9:**
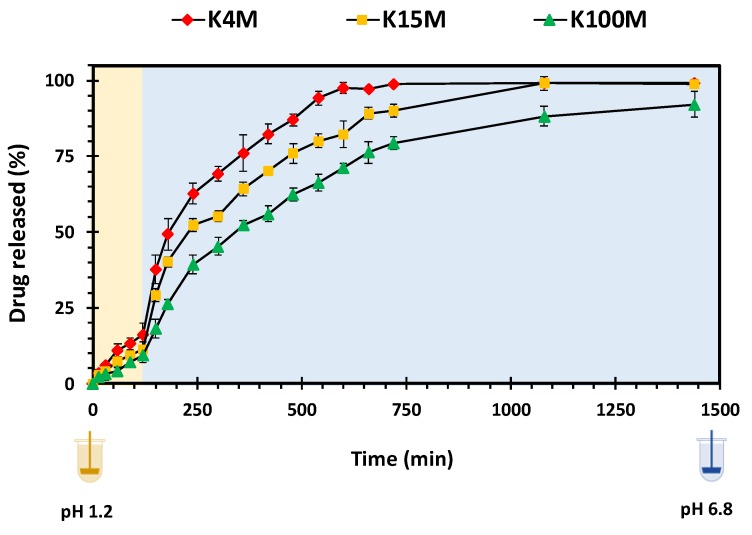
Drug (glipizide) release *vs* time profile of 3D printed HPMC matrix tablets.

Initially, HPMC forms a gel on the surface of the matrix tablets after getting hydrated on contact with the dissolution medium. Progressive contact with the medium leads to subsequent bulk hydration of the matrix. With time, the hydration results in chain relaxation of HPMC, followed by erosion of the matrix. Matrix swelling, diffusion of drug through gel layer or matrix erosion are the factors that control the drug release rate and mechanism [42,51]. Figure 9 shows the drug release (%) from all three grades of HPMC matrices with respect to time. In the first 2 h in simulated gastric fluid (pH 1.2) the glipizide was released very slowly as expected from its limited solubility in acidic media [53]. However, as the medium was changed to simulated intestinal fluid (pH 6.8) glipizide release increased due to improved solubility [54]. It is also apparent that the molecular size of HPMC played an essential role in regulating the glipizide release from hydrophilic matrices. Among these matrices, the highest drug release and lowest drug release was observed in K4M and K100M matrices, respectively. The drug release was inversely related to the swelling rate of matrices. Therefore, K100M matrices which presented the highest swelling rate (*K_w_*), Table 6, showed slowest drug release and achieved nearly 75% drug release in 750 minutes. On the other hand, K4M matrices, which displayed the lowest swelling rate (*K_w_*), Table 6, achieved 100% drug release in 750 minutes. Moreover, K15M matrices attained more than 90% drug release at the same time. Overall, it is concluded that polymer with high viscosity leads to higher swelling rate which in turn results in slow and sustained release of the drug thus, the 3D printed tablets present similar dug release behaviour which is comparable to that of matrix tablets fabricated using conventional methods, hence, proving the applicability of 3D printing technologies in the development of hydrophilic matrices. 

#### 3.3.3. Erosion Studies 

**Figure 10:**
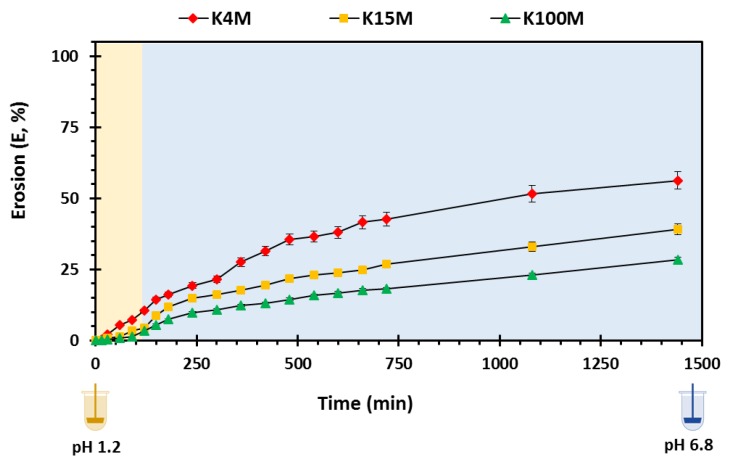
Overall matrix erosion vs, time profile of 3D printed HPMC matrix tablets.

Matrix erosion reflects the collective amount of polymer and drug dissolved [13]. The presence of polymeric carrier on the surface of hydrophilic matrices is principally responsible for the development of an outer viscous gel layer that will undergo erosion over time, and the outer gel layer controls the overall erosion rate. When the outer surface of the polymer is completely hydrated, the polymeric chains start to dissolve leading to matrix erosion. The factors that influence the erosion and drug release rate are physicochemical properties of drugs especially solubility, viscosity, chemistry, ionic strength and particle size of drug and polymer [51]. The degree of matrix erosion is shown in Figure 10 and reported as % matrix erosion (E). In the present study, the degree of erosion from the matrices increased gradually over time. However, an increase in viscosity (molar mass) resulted in a decrease in matrix erosion (K4M > K15M > K100M). Additionally, Table 6 displays the erosion kinetic parameters in which K4M matrices showed the highest rate of erosion (*K_E_*) followed by K15M and K100M matrices. The R^2^ values in the table shows a linear profile for all types of matrices confirming that matrix erosion decreased with increased molecular size (viscosity) [41,55]

### 3.4. Pharmacokinetic Parameters of HPMC/Glipizide Hydrophilic Matrices

**Figure 11:**
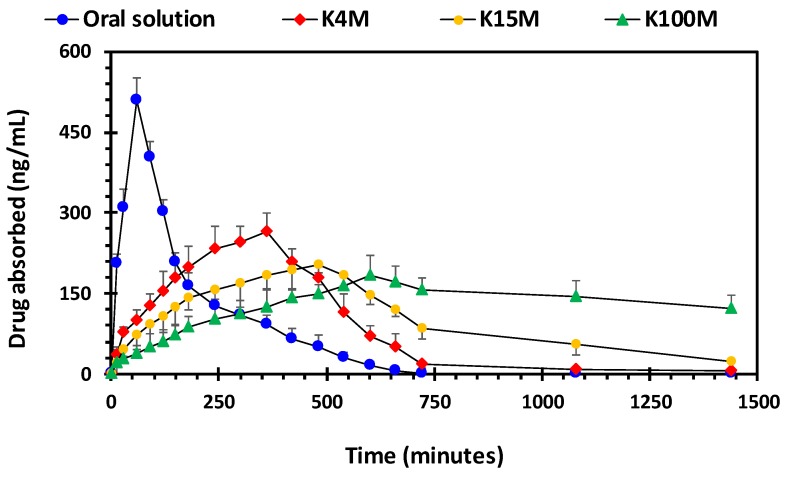
Drug (glipizide) absorption *vs* time profile of 3D printed HPMC matrix tablets.

For pharmacokinetic studies, rabbits were administered 3D printed hydrophilic matrices weighting 100 ± 1.5 mg containing 2.5% w/w glipizide. The mean plasma concentration-time curve of the printed samples and reference (oral solution) are shown in Figure 11, and the relevant pharmacokinetic parameters are given in Table 7. 

Out of all of the hydrophilic matrices, the area under the curve (AUC) of the plasma concentration (ng/ml) versus time (minutes) profile was highest for K100M matrices (3082.36 ± 251.32 ng/ml.h) followed by K15M matrices (2325.08 ± 269.40 ng/ml.h) and K4M matrices (1959.46 ± 151.32 ng/ml.h). It is evident from the findings that there is a marked difference in the AUC from different matrix tablets. Moreover, the AUC was significantly lower for the oral solution (1499.48 ± 164.65 ng/ml.h) compared with the HPMC: Glipizide matrices. This is due to the decrease in drug absorption after reaching *T_max_* (60 min). After approximately 400 min, drug absorption from the oral solution was significantly less than absorption from the HPMC: Glipizide matrices (Figure 11). Furthermore, the highest maximum plasma concentration (*C_max_*) was achieved by the oral solution (509.17 ± 42.19 ng/ml) followed by the K4M matrices (264.88 ± 33.69 ng/ml), K15M matrices (202.85 ± 15.33 ng/ml) and the lowest was attained by K100M matrices (182.66 ± 38.37 ng/ml). Although, the time taken to reach *C_max_* (*T_max_*) followed the opposite trend with the longest time being taken by K100M matrices (600 min) and the shortest time being taken by the oral solution (60 min). These pharmacokinetic parameters may be explained by the extended release effect of HPMC which causes the drug to be absorbed into the bloodstream over a longer period compared with the oral solution, thus confirming the sustained release nature of the formulations. Additionally, the reduced drug release rate caused by the higher viscosity gel formed by, the higher molar mass HPMC grades further increases the *T_max_* and reduces *C_max_*.

## 4. Conclusions

The current study has successfully demonstrated the fabrication of HPMC: Glipizide filaments and 3D printed matrix tablets without the addition of plasticiser. The XRD spectra of glipizide and drug-loaded HPMC filaments were consistent with the DSC profiles; thus, both methods showed that glipizide has a crystalline structure, whereas HPMC and glipizide loaded filaments are amorphous in nature. TGA confirmed the onset of degradation for both drug and filaments was above the operating temperature (170 ˚C) involved in the printing process. The mechanical properties of glipizide loaded filaments were robust and were successfully employed to 3D print matrix tablets. It can be concluded that the 3D printed matrix tablets were considerably rougher than the conventional compressed tablets and the current research findings have suggested that more attention is necessary to comprehend this research area. Furthermore, it can be concluded that the viscosity of HPMC has a noticeable impact of the swelling, erosion, HPMC dissolution, drug release and pharmacokinetic properties. The highest viscosity grade (K100M) tends to have a higher degree of swelling, decreased HPMC dissolution, low matrix erosion, decreased drug release and extended drug absorption profile. Overall, this study confirmed the successful fabrication of 3D printed matrix tablets which have functionalities analogous to matrix tablets fabricated using conventional technologies. Moreover, the current study has also demonstrated the usefulness of the FDM technique, providing a simple solution to develop personalised pharmaceutical formulations in a time and cost-effective manner addressing challenges confronted by conventional manufacturing processes.

## Figures and Tables

**Table 1:** Specifications of different hydroxypropyl methyl cellulose (HPMC) grades used in this study.

Methocel®	Viscosity (cps) ^a^	Average Molar Mass g/mol ^a^
**K4M**	4351	~88000
**K15M**	17129	~125000
**K100M**	79279	~215000

**^a^** Data obtained from manufacturer.

**Table 2:** Parameters used for the 3D printing of HPMC/glipizide matrices.

Printing Temperature °C	Number of Shells	Layer Height (mm)	Raft	Support	Infill Density (%)	Speed While Extrusion (mm/sec)	Speed While Traveling (mm/sec)	Infill Pattern
170	2	0.1	No	No	100	90	150	Linear

**Table 3:** Drug loading and three-point bending results of filaments (* n = 10, standard deviations are in parenthesis).

Characteristics	K4M	K15M	K100M
Drug loading efficiency (%)	98.7 (2.2)	101.2 (1.1)	99.3 (1.6)
Force (N)	1.9 (0.2)	2.2(0.6)	2.3 (0.3)
Distance (mm)	4.9 (1.2)	5.1 (1.1)	5.2 (1.4)
Stress (MPa)	12.3 (1.1)	14.2 (1.2)	16.9 (1.6)
Strain	0.8 (0.2)	0.9 (0.2)	0.80 (0.1)
Young modulus, E (MPa)	14.7 (1.2)	17.1 (1.4)	21.1 (1.6)

**Table 4:** Geometrical and morphological characteristics of 3D printed hydrophilic matrices (n = 5, standard deviations are in parenthesis).

Characteristics	K4M	K15M	K100M
Weight (mg)	100.1 (2.2)	99.6 (1.8)	99.3 (2.1)
Diameter	3.0 (0.1)	3.0 (0.1)	3.0 (0.1)
Thickness (mm)	1.5 (0.1)	1.5 (0.1)	1.5 (0.1)
Drug loading in tablets (%)	99.3 (2.6)	100.3 (2.9)	98.6 (1.4)
Porosity (%)	2.2 (0.2)	1.5 (0.2)	0.8 (0.1)
Breaking strength of tablets (N)	345.6 (10.3)	480.2 (14.6)	525.3 (9.3)
Friability (%)	0	0	0

**Table 5:** 3D quantitative surface texture parameters of HPMC/glipizide hydrophilic matrices (n = 10, standard deviations are in parenthesis).

Parameter	K4M	K15M	K100M
Sa (μm)	22.6 (2.3)	14.6 (1.9)	16.4 (3.3)
Sq (μm)	26.5 (9.4)	20.3 (3.5)	23.7 (4.8)
Sz (μm)	75.0 (7.2)	60.0 (9.1)	70.0 (10.9)
Sp (μm)	33.7 (6.8)	30.7 (4.9)	36.3 (4.0)
Sv (μm)	41.3 (9.0)	29.4 (5.3)	33.7 (8.4)
Sku (μm)	5.3 (1.1)	4.9 (0.9)	5.1 (1.4)
Ssk (μm)	−0.6 (−0.1)	−0.4 (−0.2)	−0.7 (−0.2)
Sds (1/μm^2^)	112.4 (11.4)	98.3 (5.6)	104.1 (9.7)
Sal (μm)	136.4 (20.4)	111.0 (11.0)	121.4 (9.0)
Str	0.9 (0.1)	0.9 (0.1)	0.8 (0.1)
Vm (μm^3^/μm^2^)	0.4 (0.1)	0.3 (0.09)	0.4 (0.1)
Vv (μm^3^/μm^2^)	6.9 (2.5)	5.2 (2.89)	6.4 (3.2)
Vvv (μm^3^/μm^2^)	0.6 (0.1)	0.4 (0.1)	0.3 (0.1)

**Table 6:** Swelling and erosion kinetics parameters of HPMC/glipizide hydrophilic matrices (n = 5).

Type of Matrix Tablet	Swelling Kinetic Parameters			Erosion Kinetic Parameters	
	K*_W_* ^a^	*n*	R^2^	K*_E_* ^b^	R^2^
**K4M**	32.96	0.34	0.99	0.076	0.98
**K15M**	52.20	0.28	0.97	0.057	0.96
**K100M**	76.51	0.24	0.98	0.039	0.97

^a^ Swelling rate, *K_w_* (% min^−1^); ^b^ Erosion rate, *K_E_* (% min^−1^).

**Table 7:** Pharmacokinetic parameters of hypromellose/glipizide hydrophilic matrices (n= 5, standard deviations are in parenthesis).

Parameters	K4M	K15M	K100M	Oral Solution
**T _max_ (h)**	6 (0.00)	8(0.00)	10 (0.00)	1 (0.00)
**C _max_ (ng/mL)**	264.88 (33.69)	202.85 (15.33)	182.66 (38.37)	509.17 (42.19)
**AUC_o-t_ (ng/mL.h)**	1959.46 (151.32)	2325..08 (269.40)	3082.36 (252.32)	1499.48 (164.65)
